# Cure Rate Following Rejection in Bilateral Corneal Grafts for Keratoconus

**Published:** 2010-07

**Authors:** Mitra Rahimzadeh, Ebrahim Hajizadeh, Sepehr Feizi

**Affiliations:** 1 Department of Biostatistics, Tarbiat Modares University, Tehran, Iran; 2 Ophthalmic Research Center, Labbafinejad Medical Center, Shahid Beheshti University of Medical Sciences, Tehran, Iran

**Keywords:** Penetrating Keratoplasty, Keratoconus, Corneal Graft Rejection, Cure Rate, Frailty Model

## Abstract

**Purpose:**

To estimate cure rate following graft rejection in bilateral corneal transplants in Iranian patients with keratoconus and to determine risk factors associated with rejection.

**Methods:**

In this retrospective study, data were compiled from records of patients who had undergone bilateral penetrating keratoplasty (PK) for keratoconus between 1988 and 2007. In order to estimate cure rate in patients with and without corneal vascularization, we adopted the cure rate frailty model with a Bayesian approach.

**Results:**

Two hundred and thirty-eight eyes of 119 patients underwent bilateral corneal transplantion for keratoconus, of which 22.7% experienced graft rejections. Cure rates for patients with and without corneal vascularization were 41% and 79%, respectively. Cure rate decreased 12% per decade of increase in recipient age. The 1, 5, and 10-year survival of corneal transplants without any graft rejection episodes were 82%, 74%, and 70% respectively.

**Conclusion:**

The most important risk factor predisposing to rejection in patients undergoing bilateral PK for keratoconus was corneal vascularization. Cure rate for patients without vascularization was high in this data set, indicating that penetrating keratoplasty in keratoconus patients without vascularization is an efficient and reliable procedure.

## INTRODUCTION

Corneal transplantation aims to replace abnormal corneas with normal tissue. Keratoconus is one of the most common indications for corneal transplantation, accounting for 11% to 31% of cases of penetrating keratoplasty (PK).^1^–^5^ In Iran, a total of 19,668 corneal transplants were performed between 1994 and 2004, of which, 34.5% were for keratoconus.^6^

Keratoconus is a progressive, non-inflammatory, bilateral, but asymmetrical disease in which the cornea bulges and takes an irregular conical shape instead of the normal spherical shape, resulting in myopia and irregular astigmatism and hence, poor vision.^7^–^9^ In advanced cases where contact lenses and intracorneal rings are inappropriate or not sufficient to correct vision or when contact lens intolerance develops, corneal transplant surgery may become necessary.

Keratoconus is one of the most common indications for corneal transplant procedures with a favorable outcome.^10^–^13^ However, graft failure is possible, the major cause of which is graft rejection.^14^–^17^ Approximately 6,000 corneal transplant procedures are performed each year around the world, one-third of which become complicated by graft rejection reactions at least once ultimately leading to graft failure in 5% to 7% of cases.^18^–^20^ Various studies around the world have reported different rates of rejection. Possible reasons for this discrepancy are diversity in definitions for graft rejection reactions, different durations of follow-up and various indications for transplantation.^18^

This study was designed to determine risk factors for graft rejection in keratoconus patients who had undergone bilateral PK. In keratoconus patients, the more severely affected eye is usually considered for keratoplasty, while the other is managed conservatively using spectacles or rigid gas-permeable contact lenses. In cases requiring bilateral keratoplasty, the second operation is postponed until all sutures are removed from the operated eye. We considered a corneal graft as rejected when it remained clear for at least 10 to 14 days following surgery and then started showing signs of rejection due to recipient immune response.

There are some known risk factors predisposing to corneal graft rejection, including the genetic composition of the host which is the same for fellow eyes in bilateral cases and may cause a correlation in the survival of bilateral grafts. Due to such immeasurable risk factors, utilizing the Cox proportional hazard model^21^ produces biased results.^22^ This issue can be addressed by multiplying a random effect with the hazard function. In the context of survival analysis, this random effect is referred to as the frailty effect and the adopted model is called the Cox frailty model.^23^–^25^ In the case of bilateral corneal transplantations, it is clear that data from the two eyes cannot be considered to be independent. The frailty model explicitly formulates the nature of the underlying dependence structure.^26^

To assess the goodness of fit of the models, the Deviance Information Criteria (DIC) was employed, as a result of which the smallest value shows better fitness of the model.^27^

## METHODS

Among organ transplantations, corneal transplantation has the most favorable prognosis with a clear graft survival rate exceeding 90% over a 5-year period.^28^ From a statistical point of view, these individuals are considered “cured” or immune and are not at risk for event occurrence. Although clinical observations indicate that graft rejection can occur even several decades after transplantation, the probability of graft rejection will be very small with such long-term follow up. In such situations, the population is divided into two groups. The first group consists of individuals who with adequate follow-up will eventually experience the event of interest, whereas the second group comprises individuals who are immune to the occurrence of the event. In medical studies that concern a proportion of individuals who are immune to the event of interest, analyzing data using cure models has been considered. For the first time, cure models were introduced in 1949 by Boag^29^ and later improved by Chen et al^30^ in 1999 using a Bayesian approach. Given the bilaterality of corneal transplantations, low risk of graft rejection, and the presence of unidentified risk factors influencing survival periods, we used a cure rate frailty model with a Bayesian approach^31^ to assess factors associated with rejection of bilateral corneal grafts.

In this retrospective analytic case series, records of 119 keratoconus patients who had undergone bilateral corneal transplantation at Labbafinejad Medical Center, Tehran, Iran between 1988 and 2007 were compiled. Graft survival period was considered the time interval between corneal transplant surgery and the onset of an episode of graft rejection reaction. If the eye did not experience any rejection episodes, the time interval between corneal transplantation and last examination was defined as censored survival time. Collected data included: date of surgery, date of the first rejection episode, date of final eye examination, and status of graft rejection. Recipient information included sex, age at the time of surgery, presence of severe eye allergies and vascularization of the cornea. Donor information included time interval from tissue harvesting to transplantation. If a survival function curve by the Kaplan-Meier method leveled out before reaching the zero point, it was concluded that the risk of ongoing rejection episodes was low.

Some patients with bilateral corneal grafts are not at risk of failure, and there is heterogeneity among survival times, in order to account for these problems, the cure rate frailty model with a Bayesian approach by Yin^31^ was applied for the estimation of the prognostic factors of corneal graft rejection.

Since piecewise exponential models are flexible and fit well in the hazard function of any type of data, this function was used. In addition, the log-normal probability distribution function was utilized for frailty distribution.

## RESULTS

**Figure 1:**
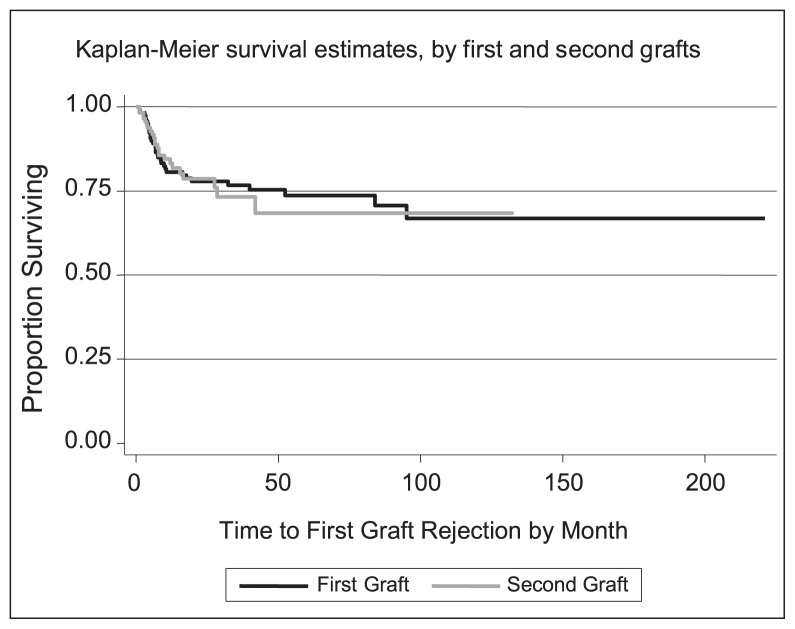
Kaplan Meier survival curves based on first and second grafts.

Overall 238 eyes of 119 subjects with keratoconus underwent bilateral PK and were followed for a mean of 43.9 months (range, 1–221 months). During the follow-up period, 54 patients (22.7%) experienced first graft rejections, 31 of which involved the first eye and the rest happened in the second eye. Overall, 18 cases of corneal graft rejections were bilateral (9 patients) and 36 cases were unilateral. Rejection episodes occurred 1.1 to 95 months postoperatively with a mean and median of 12.5 and 6.9 months, respectively. Rejection occurred in 20.8% and 58.3% of patients without and with corneal vascularization, respectively. No statistically significant difference was found between graft survival in first and second eyes using the Log rank test (p=0.9). Based on the estimation of parameters in the cure frailty model, patient age and presence of corneal vascularization had a significant negative correlation with cure rate (P < 0.05; table 1). The 1, 5, and 10-year rejection-free survival rates of corneal transplants in this study were 82%, 74%, and 70%, respectively (Fig. 1).

Given that rejection episodes are most likely to occur in the first 18 months following surgery, a small or moderate number of intervals is recommended in cure models.^32^ We divided the follow-up period into two time spans of 0 to 18 months, and 18 to 221 months to estimate piecewise exponential hazard functions which revealed that the risk of graft rejection in the first time span is 6 times higher than that in the second time span, resembling other studies.^33^ Using more intervals, we observed that the DIC increased (table 2).

**Figure 2:**
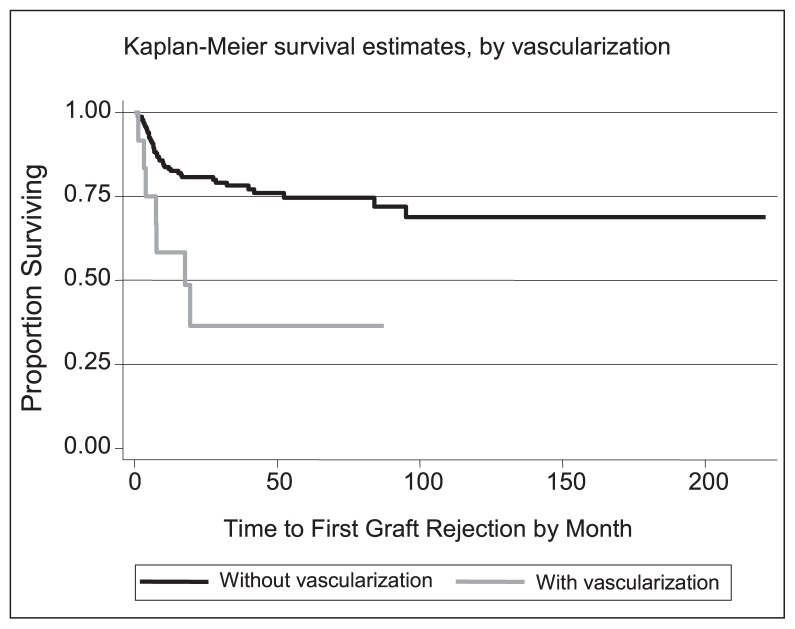
Kaplan Meier survival curves based on the presence or absence of vascularization.

The frailty effect variance > 0 indicates the presence of other unknown risk factors which cannot be addressed using available covariates. In the cure model, cure rate with and without vascularization for example is calculated using the following formula: exp (−exp (intercept + beta × parameter)). Thus, using the cure rate frailty model in this study, cure rate was estimated to be 41% for patients with vascularization and 79% for patients without vascularization (Fig. 2). Cure rate was reduced by 12% per decade of increase in recipient age.

We used the Cox frailty model that assumes all subjects will experience the event if follow-up is extended (results not shown). The DIC determined both models and its smallest value showed better fit for cure frailty models. Therefore, the cure or immunity rate is significant in this data set (table 2).

In cases with repeat transplantations, the time interval between the first and second graft was 3.8 to 204 months with a mean of 44.6 months. This period was less than a year in 7% of cases who underwent regrafts. In order to assess the effect of time interval between the two transplant surgeries, we used the cure model and incorporated time interval into the model as an influencing factor. Based on this model, cure rate for the second eye was significantly reduced by decreasing the time interval from the first to second transplants (P=0.01).

## DISCUSSION

Identification of risk factors affecting cure rates and estimation of rejection-free survival rates in subgroups of corneal transplant patients are of great importance. Corneal transplantation is one of the most successful organ transplantation procedures. However, corneal graft rejection is relatively common and is the leading cause of corneal graft failure, resulting in graft failure in 30% to 40% of patients.^34^ In this study, recipient corneal vascularization and advanced recipient age were found as risk factors influencing cure rate. Rejection occurred in 20.8% and 58.3% of the patients without and with corneal vascularization, respectively. A study by Khodadoust and Karnema^35^ revealed a rejection rate of 7.5% and 39% in non-vascularized and vascularized corneas, respectively (P<0.05).

During the follow-up period, rejection occurred in 22.7% of subjects in the present study which is comparable to the rate (26.6%) reported by Sinha et al^36^. The rate of graft rejection was 26.1% and 19.3% for first and second eyes, respectively and exceeds the rates (11% and 8%) reported by Rao et al.^37^ The higher rejection rate for the first eye as compared to that for the second eye can be attributed to the longer follow-up period. However, the difference between the two eyes did not reach a significant level in either of the studies. This observation is in line with the results of Ozbek et al.^38^

Different studies report varying outcomes after bilateral corneal grafts. In this study, decreasing the time interval between the first and second transplants resulted in reduction of cure rate. Buxton et al^39^ noted that performing a corneal transplant on the second eye is a risk factor for graft rejection in the first eye, whereas in the study carried out by Donshik et al^40^ a corneal graft in the second eye did not impose any threat to the first.

Using multivariate analysis of data obtained from bilateral corneal transplants, we assessed factors influencing cure rate. In this study, graft survival durations were considered as parallel variables. However, this is not the case in reality and it must be noted that the transplant on the second eye was performed after a period of time following transplantation in the first eye. Additionally, this study is retrospective, therefore, it is not possible to systematically review all risk factors such as loose sutures and previous ocular surgery as some data were missing and certain patients were lost to follow-up.

## Figures and Tables

**Table 1:** Parameter estimation in the cure rate frailty model

Parameter	Mean	SD	P-value
Intercept	−1.45	0.297	0.0001
Age	0.05	0.017	0.01
Vascularization	1.34	1.067	0.00001
Lambda 1*	0.064	0.012	0.001
Lambda 2*	0.016	0.01	0.005
Frailty	1.8	0.68	0.001

SD, standard deviation

* parameter of piecewise distribution

**Table 2:** Model selection criteria based on DIC with respect to time spans

	Interval (months)	
	
	(0,18] (18,221]	(0,6] (6,18] (18,60] (60,221]
	
Cox Frailty Model	449.19	452.36
Cure Frailty Model	431.51	435.68

DIC, deviance information criteria

## APPENDIX A

If the variable names were:

Age: Continuous

Vascularization: Categorical (1 if with vascularization, and 0 if without vascularization)

The probability of cure was:

π=exp (-exp(-1.45+1.34 (Vasc)+0.05 (Age)))

Where:

The probability of cure in patients with vascularization conditional of age was:

π=exp (-exp(-1.45+1.34))

and that of subjects without vascularization was:

π=exp (-exp(-1.45))
